# Designing an Indoor Radon Risk Exposure Indicator (IRREI): An Evaluation Tool for Risk Management and Communication in the IoT Age

**DOI:** 10.3390/ijerph18157907

**Published:** 2021-07-26

**Authors:** Sérgio Ivan Lopes, Leonel J. R. Nunes, António Curado

**Affiliations:** 1 ADiT-Lab, Instituto Politécnico de Viana do Castelo, Rua da Escola Industrial e Comercial de Nun’Alvares, 4900-347 Viana do Castelo, Portugal; 2 IT—Instituto de Telecomunicações, Campus Universitário de Santiago, 3810-193 Aveiro, Portugal; 3 PROMETHEUS, Unidade de Investigação em Materiais, Energia e Ambiente para a Sustentabilidade, Escola Superior Agrária, Instituto Politécnico de Viana do Castelo, Rua da Escola Industrial e Comercial de Nun’Alvares, 4900-347 Viana do Castelo, Portugal; leonelnunes@esa.ipvc.pt; 4 PROMETHEUS, Unidade de Investigação em Materiais, Energia e Ambiente para a Sustentabilidade, Escola Superior de Tecnologia e Gestão, Instituto Politécnico de Viana do Castelo, Rua da Escola Industrial e Comercial de Nun’Alvares, 4900-347 Viana do Castelo, Portugal; acurado@estg.ipvc.pt

**Keywords:** radon gas, IoT, visual analytics, risk analysis, risk mitigation, risk management, risk communication, risk public perception

## Abstract

The explosive data growth in the current information age requires consistent new methodologies harmonized with the new IoT era for data analysis in a space–time context. Moreover, intuitive data visualization is a central feature in exploring, interpreting, and extracting specific insights for subsequent numerical data representation. This integrated process is normally based on the definition of relevant metrics and specific performance indicators, both computed upon continuous real-time data, considering the specificities of a particular application case for data validation. This article presents an IoT-oriented evaluation tool for Radon Risk Management (RRM), based on the design of a simple and intuitive Indoor Radon Risk Exposure Indicator (IRREI), specifically tailored to be used as a decision-making aid tool for building owners, building designers, and buildings managers, or simply as an alert flag for the problem awareness of ordinary citizens. The proposed methodology was designed for graphic representation aligned with the requirements of the current IoT age, i.e., the methodology is robust enough for continuous data collection with specific Spatio-temporal attributes and, therefore, a set of adequate Radon risk-related metrics can be extracted and proposed. Metrics are summarized considering the application case, taken as a case study for data validation, by including relevant variables to frame the study, such as the regulatory International Commission on Radiological Protection (ICRP) dosimetric limits, building occupancy (spatial dimension), and occupants’ exposure periods (temporal dimension). This work has the following main contributions: (1) providing a historical perspective regarding RRM indicator evolution along time; (2) outlining both the formulation and the validation of the proposed IRREI indicator; (3) implementing an IoT-oriented methodology for an RRM indicator; and (4) a discussion on Radon risk public perception, undertaken based on the results obtained after assessment of the IRREI indicator by applying a screening questionnaire with a total of 873 valid answers.

## 1. Introduction

Radon gas (Rn-222) is a common pollutant in indoor air that was classified as a carcinogenic agent to humans by the International Agency for Research on Cancer (IARC) as part of Group 1 and as a Group A carcinogen by the US Environmental Protection Agency (US EPA) [1,2,3]. Despite other perceptions, such as those described in several studies, and such as those presented by Cohen (1995), Becker (2004), Thompson et al. (2008), Dobrzyński et al. (2018), or even Pylak et al. (2021), where the authors differed with the generalized opinion about the connection between low Radon concentration and lung cancer [4,5,6,7,8], many research demonstrations that the inhalation of Radon and its decay products may impact human health triggering a potential increase in lung cancer risk can be found [9]. Indoor Radon exposure is widely considered the second-largest risk factor associated with lung cancer [10]. Arising as a by-product resulting from the decay sequence of the uranium (U-238), a chemical element present in soils and rocks, Radon is directly formed from Radium (Ra-226) decay, another radioactive element resulting from the Uranium decay chain, on which alpha radiation particles are emitted [11]. Radon is a radioactive gas detected in soils and rocks that can easily make its path through the cracks of stones, bedrock, and soils, moving into the outdoor atmosphere where its dilution is almost immediate, or into indoor environments, where it can accumulate and reach high concentration levels, therefore causing the risk of the occupants to be exposed [12,13,14]. Despite Radon being a natural element of breathing air, the exposure of people to Radon both at home and at work must be correctly managed by using a straightforward methodology which is easy to apply [15]. 

In contrast to other radioactive elements, Radon is the single radioisotope that occurs in nature in a gaseous state [16]. Due to its high mobility, Radon diffusion arises from local environments with higher concentrations to others with lower concentrations [17]. However, Uranium minerals occur in nature in different local concentrations and forms, so Radon concentration differs worldwide [18]. The formation of Radon occurs in the superficial soil layer, and the gas spreads throughout its interstices to the surface, using the pressure difference between outdoor air and the soil gaseous fraction that holds Radon [19]. The gas that flows from the soil to the outdoor air presents no risk to human health, given the dilution effect. However, the gas that flows into buildings through the foundations or cracks in the ground level pavement increases the Radon exposure risk to the occupants [20]. When Radon gas enters into an enclosed space due to an air pressure gap between indoor air and soil, multiple paths for the propagation of the gas can be found: pipes and drains, structural joints, walls and foundations, construction cracks, sumps, and holes, among others [21]. Once inside the enclosed space, Radon tends to accumulate in lower levels (because it is approximately 10 times heavier than air) [22], its removal being normally based on manual or mechanical ventilation techniques that promote air renovation [23]. However, despite ventilation, which is relevant for Radon risk mitigation, geology plays a decisive role in the appearance of Radon gas on the surface of the Earth [24]. In general, the nature and permeability of the soil, the porosity of the rocks, and the presence of cracking horizons favor Radon transfer into buildings [24]. This explains why a house built on granite soil, for example, can have a Radon concentration several times higher than another located on sedimentary soil [25]. Soft soils, dominated by layers of sand and clay, contain relatively low Radon contents [26]. Clay layers can even prevent Radon from migrating to the surface [26]. On the other hand, hard rocks, such as granites, limestones, sandstones, and shales, may contain relatively high Radon contents [27]. 

Despite the importance of soil geology in indoor Radon concentration, buildings supported by foundations laying on the granitic bedrock or settled on volcanic areas, both prominent in Uranium, are more likely to have high Radon indoor concentration [28]. The time that people spend indoors worldwide is growing rapidly, mainly during winter or in regions with severe weather conditions, where people spend most of their time inside badly ventilated enclosures to avoid hard weather conditions, and therefore increasing Radon risk exposure [29]. On the other hand, the common use of different types of heating and air conditioning solutions determines less air renovation by natural ventilation, thus contributing to potentiate indoor Radon exposure [30]. In combination with a less ventilated built environment, indoor Radon exposure is therefore strongly related to the occupancy of the buildings [31].

Statistics show that throughout Europe about 90 million youngsters and infants spend their weekly time at school, 33% of the active population work in offices, and about 90 million sick people spend every year at least one week in hospitals or clinics. Overall, Europeans spent 90% of their time indoors, not only at home but also working, at schools or hospitals [32], thus increasing lung cancer risk, due to the long-term cumulative exposure to indoor Radon gas, in areas with high Radon potential. Despite there existing many other risk factors for lung cancer related to environmental, behavioral, societal, economic, and biological factors, the cumulative effect of smoking habits and long-term Radon exposure presents a considerable increase in lung cancer risk [33,34,35]. The literature stresses an amplification of the risk when Radon exposure is combined with smoking habits (86% of Radon lung cancer deaths occurred in former smokers) [36,37]. Given all these variables, the study of Radon concentration over time is a complex subject, dependent on multiple factors, and the indoor concentration average should be interpreted strictly as a statistical quantity. In reality, sometimes large differences within the same region or in two adjoining buildings can be observed. Experimental results attest that Radon distribution is presented as a discrete case, hence the need to carry out measurements in each building [38].

Currently, several IoT-based Radon monitoring architectures are being proposed [39,40,41,42,43,44,45]. Typically, these devices are designed for online monitoring, i.e., Radon levels are continuously collected and communicated to a cloud for further analytics, and after reasoning, specific mitigation actions can be performed. However, no risk indicator can be used in a continuous and online context based on the average Radon gas concentration in a specific period or on the effective exposure of users for a given time (occupancy). In addition, poor Radon risk communication can lead to a misperception of the risk and therefore failure to produce the pretended risk reduction among the population. Therefore, new visual analytical approaches must be put forward for effective Radon risk perception [46].

This work introduces the IRREI (Indoor Radon Risk Exposure Indicator), a tool designed to increase the Radon risk communication effectiveness, that consists of an objective risk scale, that can be used in IoT-based architectures to enhance web applications with rich visual analytics [46]. Moreover, the pervasiveness of IoT technologies for IAQ monitoring [47,48,49] can also be useful for Radon Risk Management (RRM), since active mitigation strategies can also be integrated within the IoT ecosystem in domains such as smart buildings [40]. However, data visualization and intelligibility demand the definition of relevant metrics and key risk indicators, which may be computed upon continuous real-time data. The approach here presented is focused on the design of an intuitive Indoor Radon Risk Exposure Indicator (IRREI), specifically designed for decision-makers, building managers, or just for the ordinary citizen, by taking advantage of IoT-based data availability and supported on continuous data with specific spatial–temporal attributes. The metrics proposed here include relevant factors from three core domains, such as the ICRP dosimetric limits (regulatory dimension), building occupancy (spatial dimension), and exposure periods (temporal dimension). 

The main contributions of this work include the following tasks: (1) to outline a historical perspective regarding RRM evolution over time; (2) to define and evaluate the proposed IRREI; (3) to propose a new IoT-oriented methodology for RRM. Lastly, a discussion is undertaken based on the results obtained after evaluation of the IRREI using an online survey with a total of 873 valid responses. The remainder of this article is organized as follows: Section 2 presents a historical perspective about RRM; Section 3 presents the materials used, and the methodology adopted during the research; Section 4 presents and discusses the results achieved, i.e., the proposed IRREI and the IoT-oriented methodology for RRM; and lastly, in Section 5, the conclusions are outlined.

## 2. Radon Risk Management—A Historical Perspective 

### 2.1. Framework

**Figure 1:**
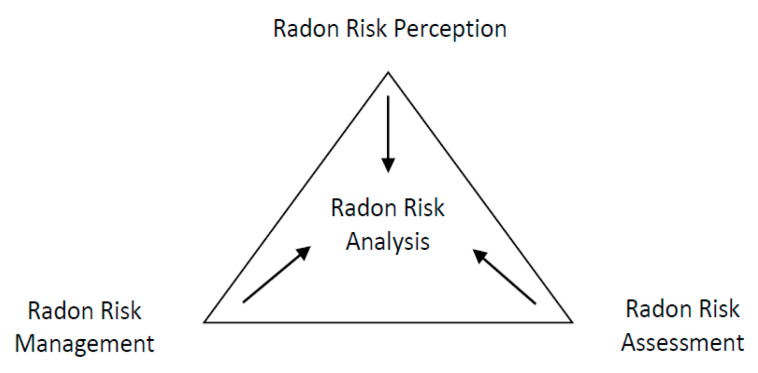
Radon risk analysis.

Radon risk analysis requires a multidisciplinary approach, using different fields of expertise. Schematically, according to Figure 1, three complementary perspectives from different angles, including risk perception, risk assessment, and risk management, can be identified. According to each specialization field, Radon risk assessment concerns mostly the fields of physics, chemistry, and biology, while Radon risk perception is, above all, focused on the fields of sociology, ethnology, and psychology, and Radon risk management respects, among other things, the fields of medicine, engineering, and politics. In practical terms, Radon risk perception is related to the evaluation of which populations segments are most aware of Radon hazards and threats [50], while Radon risk assessment is focused on Radon testing and mitigation [51]. From a more global perspective, Radon risk management involves a comprehensive strategy for reducing exposure to Radon gas, including testing and remediation techniques when necessary [52].

From a historical perspective, the study of Radon risk exposure took its first steps through the coordinated action of the International Commission on Radiological Protection (ICRP), in the late 50s of the 20th century, among the communities of Uranium miners, and evolved from then on. Before 1958, the ICRP produced some recommendations regarding the entire body and exposure of body extremities to Radon gas, throughout 1928 and 1934 [53,54,55,56]. Despite this vision lasting until 1958, over the past century three main approaches have been used by the ICRP to manage Radon risk exposure: the dosimetric approach, the epidemiological approach, and more recently an integrated approach focused on the protection of occupants for all types of buildings to indoor Radon exposure, regardless of their use and type of occupancy [57]. Section 2.2, Section 2.3 and Section 2.4 synthesize the evolution of Radon risk management since the initial concerns back to the 50s of the 20th century up until today.

### 2.2. From 1958 to 1993—The Dosimetric Approach

The dosimetric approach was the first used by the ICRP regarding the work of miners in the Uranium mining process. The main concern of this approach was to establish a limit for the allowable air Radon concentration of the Uranium mines, established to protect the lung epithelium, considered by that time (1959) as the “*critical tissue*”, according to ICRP PUB 2 [58]. Later on, in ICRP Publication 26 [59], the term “*effective dose*” was introduced to characterize the scenario when the whole body is exposed to radiation, the sensitivity of a particular part (organ or tissue) can be critical, and as such can determine the dose limitation for a certain person when subjected to radiation [59,60,61]. Within this framework, both ICRP Publication 26 and ICRP Publication 32, recommended for mine workers and workers occupationally exposed to Radon and “*Radon daughters*”, a limit of 50 millisieverts (mSv) in a complete year, for the dose-equivalent limit for the whole body. For the public, the limit was 5 mSv in a year. The International System of Units (SI) unit applied to quantify radiation dose is the sievert (Sv), by quantifying amounts like “*equivalent dose*” or “*effective dose*”. The millisievert (mSv) represents one-thousandth of an Sv, and it is a radioprotection unit that quantifies the radiation exposure dose that a person can receive not only from a natural source of radiation but also from medical sources just like X-rays. 

By that date, Radon risk exposure was assessed based on the ICRP dosimetric approach by using models of the human respiratory tract to estimate the equivalent dose to the lung and effective dose per unit of Radon exposure. Until the beginning of the 80s of the past 20th century, ICRP recommendations were mostly dedicated to the protection of miners and other workers directly exposed to Radon. Additionally, based on the concept of dose limitation for miners, ICRP Publication 24 [59] defined, in 1977, the early recommendation regarding Radon protection in mines. The referenced publication touched on issues like monitoring procedures, control activities, protection devices, and medical observation for miners. More comprehensively, The United Nations Scientific Committee on the Effects of Atomic Radiation (UNSCEAR) published in 1982, for the first time, a report identifying the sources of natural radiation for the general public and its biological effects [62]. The first ICRP publication dealing with general public exposure to Radon was published in 1984 [60,63], referring to two different types of Radon exposure situations: existing and new. The publication defined, in a general way, the use of an action level to deal with the problem and to implement a mitigation strategy. Regarding the existing situations, ICRP Publication 39 recommended the implementation of an action level involving remediation actions for values of an effective dose of 20 mSv in a year; however, the recommended limit of exposure for the public was 5 mSv in a year. For situations corresponding to future exposure, the ICRP suggested an upper limit of an individual dose of 10 mSv in a year.

### 2.3. From 1993 to 2007—The Epidemiological Approach

Since 1993, the ICRP has established a change in the target population subjected to the recommendations regarding Radon protection, by considering since then not only miners but also the general public as a priority. The evaluation of the risk directly connected to Radon exposure shifted from a dosimetric to an epidemiological point of view [64,65]. The epidemiological surveys were a direct product of the straight monitoring of population cohorts exposed to Radon. Therefore, Radon risk was evaluated by analyzing the health outcomes detected in this cohort, matched to those detected in a population without exposure. The conversion factor of the dose was obtained based on epidemiological surveys by adopting the risk ratio of lung cancer in miners to the global cancer risk in survivors of the atomic bomb. In 1993, ICRP Commission developed a complete disclosure, dedicated to Radon safeguarding by bringing in two kinds of exposure events: (1) in dwellings; and (2) at worksites [66]. ICRP Publication 65 differentiated three groups of exposure concerning Radon gas [64]: (1) workers subjected to Radon occupational exposure in mines; (2) workers taken as public members, for instance at worksites; and (3) public members occupying houses, dwellings, and apartments.

The dose conversion factors obtained from the epidemiological studies were applied to obtain the concentrations of Radon from the limits of the effective dose. The importance of Radon concentrations depended on Radon exposures both in dwellings and in workplaces. In dwellings, the exposures to Radon demand intervention, and in workplaces, besides the intervention, a continuous control for Radon exposures is needed. It is important to state that the concept of action level was resumed in ICRP Publication 65, after being originally referred to in ICRP Publication 39 [67], as a limit after which to start an intervention in dwellings, both existing or new and designed to reduce Radon risk exposure. On the other hand, in workplaces, ICRP Publication 65 differentiated the miners from the other workers who were exposed to radiation. The miners were regarded as workers who were occupationally subjected to the so-called occupational annual limit (according to ICRP Publication 60 [68]) of an effective dose of 20 mSv per year on average in the last 5 years. The other workers were considered similarly to the other members of the public exposed to radiation, and so an action level was defined to handle the Radon exposure and, so, a set of mitigation measures should be considered (annual effective dose from 3 to 10 mSv, which corresponds to a Radon concentration of 200 to 600 Bq·m^−3^, adopting an occupancy on an annual basis of 7000 h, and a value of 0.4 for the equilibrium factor, used for living areas). The SI unit concerning radioactivity measurement is the becquerel (Bq), and it can be represented considering one nuclear disintegration per second. 

For the workers and public members not subject to occupational exposure to radiation, the action level both for intervention in workplaces and dwellings was the same, achieving an effective annual dose from 3 to 10 mSv corresponding to an indoor Radon concentration of 500 to 1500 Bq·m^−3^, by taking an annual occupancy of 2000 h, and a value of 0.4 for the equilibrium factor).

### 2.4. Since 2007—The Integrated Approach

The integrated approach currently followed by the ICRP on Radon protection involves both technical and societal issues and is commonly known as “*Radon risk management*”. The technical considerations are shaped in dose limits, defined as a frontier able to anticipate the so-called deterministic effects and to restrict the stochastic effects. The deterministic effects of radiation amount to death or permanent damage as a result of high doses of radiation exceeding an exposure limit value. On the other hand, the stochastic effects of radiation are related to cancer or cell mutations. With this in mind, the ICRP recommendations regarding the protection against radiation envisage that the level of exposure to Radon gas is never low enough to be safe. Based on this principle, even a low level of exposure can produce a stochastic effect like cancer. The principle suggested by the ICRP advocates that the level of exposure should always stay below the dose limits, ensuring at the same time that the referenced level of exposure should be maintained as low as technically feasible. 

Based on this, ICRP Publication 103 [69,70] recommends that any Radon exposure should be reduced to as low as reasonably possible below the reference level, whereas previous recommendations only focused on exposures above the action level. The document also defines three Radon exposure situations which were defined as follows: the planned exposure refers to situations involving the deliberate introduction and operation of sources where radiological protection can be planned, the emergency exposure regards situations that may occur during the operation of a planned situation, or from a malicious act, or any other unexpected situation, and require urgent action to avoid or reduce undesirable consequences, and existing exposure derives from situations that already exist when a control decision has to be taken, including prolonged exposure situations after emergencies. According to this, the ICRP classified indoor Radon in dwellings and workplaces as an existing exposure situation and, as a consequence, recommended that reference levels, defined under the conditions of the so-called individual dose, must be implemented across the spectrum of the management of Radon exposure. 

The reference level is the dose above which it is judged to be inappropriate to allow exposures to occur, and below which occupants’ protection should be implemented. The ICRP underlined that, for the sake of continuity and practicability, the Commission retains the upper value of 10 mSv for the individual dose reference level. Reference levels in terms of activity concentrations of Radon derived from this effective dose are also endorsed: 600 Bq·m^−3^ for dwellings and 1500 Bq·m^−3^ for workplaces. Moreover, ICRP Publication 103 defines three types of exposure: occupational exposure, which represents an exposure of workers incurred as a result of their work; medical exposure, related to exposure of patients as part of their diagnosis or treatment, volunteers helping in the support and comfort of patients, and volunteers in biomedical research; and public exposure, regarding members of the public’s exposure rather than medical and occupational exposures, and not incorporating the regular local natural background radiation [69].

Based on epidemiological results obtained both from miners directly exposed to low Radon concentration and from research concerning lung cancer derived from indoor Radon exposure, ICRP Publication 115 actualized, in 2010, the estimations of the lung cancer risk derived from Radon exposure and its consequences [71,72]. By considering an adult population of smokers and non-smokers, this publication actualized the value of the detriment-adjusted nominal risk coefficient for lung cancer to 3 × 10^−10^ per Bq·h·m^−3^, doubling the value given in ICRP Publication 65 [64]. Bearing in mind these updates, the limit-value for the indoor Radon reference level was reduced from 600 Bq·m^−3^ to 300 Bq·m^−3^.

By keeping the same principles and methodology envisaged both in ICRP Publication 103 [69] and ICRP Publication 115 [71], as well as the same Radon risk scientific evaluation, in 2014, ICRP Publication 126 [73] came up with up-to-date recommendations concerning Radon exposure, by adopting an integrated approach for indoor Radon protection. Regardless of the type of building (residential, commercial, administrative, among others) and its occupancy schedules, the reference level referred to in ICRP Publication 115 [71], as well as the protection measures to adopt against Radon exposure, must be optimized for every circumstance. Based on that, the limit of 10 mSv annual effective dose, according to ICRP Publication 65 [64], continues to be advised upon, so therefore, the authorities must impose a new reference level that must be as low as possible (100–300 Bq·m^−3^).

In 2017, ICRP Publication 137 [74], introduced new dose conversion factors for homes and workplaces that must be used; for instance, for the determination of effective doses for workers at workplaces with increased Radon exposure. According to ICRP Publication 137, based on epidemiological models, the proposed dose conversion factor for adults is 3.3 mSv per mJ·h·m^−3^. On the other hand, based on dosimetric models, the dose coefficients for exposure are 3.3 mSv per mJ·h·m^−3^ for miners, 4 mSv per mJ·h·m^−3^ for sedentary office workers, 3.7 mSv per mJ·h·m^−3^ for homes, 6.7 mSv per mJ·h·m^−3^ for tourist caves, and 5.7 mSv per mJ·h·m^−3^ for other indoor workplaces. Both approaches show remarkable consistency between dose coefficients. Taking account of both methods, the dose coefficient presented in ICRP Publication 137 for buildings and underground mines is 3 mSv per mJ·h·m^−3^. The corresponding dose coefficient expressed in terms of Radon gas exposure depends on the equilibrium factor, F, between Radon gas and its progeny. Using the standard assumption of F = 0.4 for the majority of indoor situations, this dose coefficient corresponds to 6.7 × 10^−9^ Sv per Bq·h·m^−3^.

As is stated, the ICRP dealt with Radon risk by using epidemiological investigations involving groups of people strongly subjected to high levels of Radon gas (majorly by groups of miners). Dosimetric analysis has been a powerful tool to assess Radon risk and to evaluate its impact on human health. Since 2007, due to recent epidemiological research involving not only groups of miners but also workers and building occupants, the ICRP recognized that the indoor Radon risk had been undervalued. Since then, a new integrated approach including both epidemiological and dosimetric models has been behind all ICRP recommendations. Based on those guidelines, national authorities must set a Radon reference level in the range of 100–300 Bq·m^−3^ for Radon control in residential buildings and workplaces. To calculate the effective dose due to Radon exposure in workplaces, new dose coefficients were published in ICRP Publication 137 [74]. By considering the 2013/59/Euratom Directive reference level for all Member States of 300 Bq·m^−3^ [75,76], the corresponding annual effective dose is 4 mSv for workplaces and 14 mSv for residential buildings.

**Figure 2:**
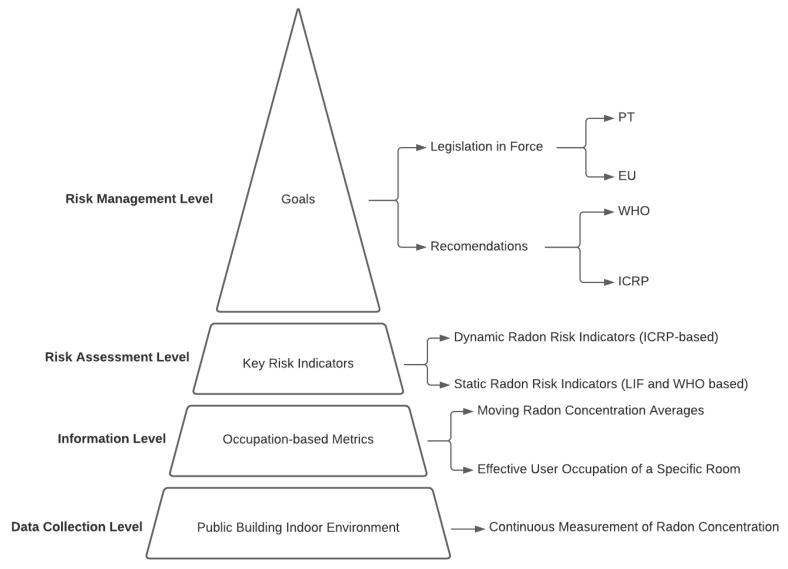
Indoor Radon risk management strategy (PT—Portugal; EU—Europe; WHO—World Health Organization; and ICRP—International Commission on Radiological Protection).

Following the referred ICRP methodology, Figure 2 shows a pyramid with a hierarchy of levels defined to establish an integrated Radon risk management strategy. The levels are the following: the data collection level; information level; risk assessment level; and risk management level. The data collection level involves the measurement of Radon concentration by using certified probes and pre-defined measurement procedures. The information level is based on the metrics calculation using all the data collected. The risk assessment level includes the definition of Key Performance Indicators (KPIs) to assess the risk. Finally, the risk management level involves the definition of a solution to solve the problem. This solution can involve a mitigation strategy, if possible, or simply the management of the occupation in a room or an enclosed space.

## 3. Materials and Methods

### 3.1. Radon Exposure and Effective Dose

The concern regarding Indoor Air Quality (IAQ) increased, given the significant number of occupants over long periods [77,78]. A large number of people remain in enclosed spaces during their working hours, with no possibility of opening windows for air renovation and depending on centralized forced air systems for ventilation and thermal comfort [79]. In such circumstances, indoor air is in general badly recirculated by undersized mechanical ventilation systems and, consequently, contaminants within buildings gradually accumulate due to the lack of air renovation [80]. Radon gas is a natural contaminant of the air that people breathe inside buildings, constituting the principal source of natural origin ionizing radiation [81]. 

To assess indoor Radon concentration, it is fundamental to estimate the concentration of Radon in buildings and dwellings [82]. Radon meters make it possible by counting the impacts of α particles and then computing the number of impacts and the duration of the measurement, to determine the concentration of Radon in the air, expressed in Bq·m^−3^ [83]. It has been found that the Radon level in buildings is also very variable. This rate varies in space (from one room to another, from one building to another), and in time (from one day to another, depending on the air circulation) [84]. A home built from granite can, for example, have a Radon concentration more than ten times higher than that measured in a house built of concrete [85]. Likewise, the season, climate, wind speed, atmospheric pressure, humidity, and temperature modify the domestic concentration of Radon [86]. This is the reason why it is recommended to measure Radon concentration for three months, during the winter, in places usually with occupancy [87]. 

The most recent recommendations of the International Commission on Radiological Protection (ICRP) suggest an integrated approach for protection against Radon exposure in buildings and suggest that national authorities should impose a reference level as low as reasonably achievable (100 to 300 Bq·m^−3^), corresponding to an upper benchmark of about 10 mSv per year (ICRP Publication 126) [73]. In 2009, the report developed by the World Health Organization (WHO) defined that the limits of exposure to Radon gas in buildings should be decreased to 100 Bq·m^−3^ [88].

**Figure 3:**
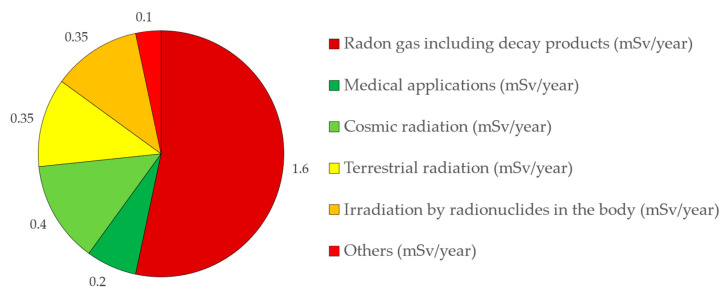
Most representative origins of exposure of the world population to ionizing radiation (adapted from [91]).

Most people face radiation exposure in their daily lives without being aware of it, comprehending exposure to external natural radiation from sideral space and the Earth, and exposure to internal naturally present radioactive materials, just like those in food and indoor Radon [2]. The principal source of radiation from ionizing sources with natural origins is Radon gas and the subsequent progeny, referred to as its decay products [89]. According to the United Nations Scientific Committee on the Effects of Atomic Radiation (UNSCEAR), the inhalation of Radon and progeny elements corresponds to a dose along the year that represents most of the exposure to natural origins [90]. According to the graph presented in Figure 3, Radon gas including its decay products is the most representative origin of exposure of the world population to ionizing radiation [91]. Overall, natural radiation represents about a 60% annual dose that affects people, and medical practices represent about 40% of the remaining dose.

When radiation passes through human organs and tissues, damage occurs within the cells and DNA molecules, which have self-protection processes to fix damage. Nevertheless, if poorly repaired this may be the beginning of a complex multistage action that can reach cancer occurrence. According to UNSCEAR, the dose is the absorbed energy as a consequence of radiation exposure, which is accounted for in three distinct forms: the absorbed, the equivalent, and the effective dose. The embedded energy in the human organism derived from radiation exposure is called the absorbed dose, the biological health effect concerning radiation alpha, beta, and gamma is the equivalent dose, and the effective dose is an indicator that signs the effect of radiation exposure on health, obtained by the multiplication of the equivalent dose by a factor related to tissue weighting for a specific organ or tissue.

Concerning Radon gas exposure, whenever someone inhales air containing Radon, the calculation of the effective dose is made by the use of the following data: indoor Radon concentration determined by in situ measurement; time of exposure to the referenced concentration; and the factors for dose conversion. The calculation of radiation exposure is made based on hours of work, and the in-situ results are generally obtained from workplaces, or working environments, so that exposure scenarios can be simulated. Consequently, indoor Radon exposure is obtained by the multiplication of the indoor Radon concentration by the time passed under this concentration, in Bq·m^−3^·h. For example, the effective dose received by an employee derived from exposure of 1 Bq·m^−3^·h is 6.7 × 10^−9^ Sv, considering an equilibrium factor of 0.4. If for the measurement period the employee has labored for 300 h exposed to an indoor Radon concentration of 500 Bq·m^−3^, and for 100 h exposed to a concentration of 1000 Bq·m^−3^, the total exposure of the employee is (300 h × 500 Bq·m^−3^) + (100 h × 1000 Bq·m^−3^) = 250,000 Bq·m^−3^·h. Under these conditions, the employee has received a dose for the measurement period of 250,000 Bq·m^−3^·h × (6.7 × 10^−9^ Sv (Bq·m^−3^·h)) = 0.0017 Sv = 1.7 mSv. 

The radiation exposure of the employees must be provided when the action level is overtaken, even when remediation procedures were implemented to control exposure. The aim of measuring radiation exposure is to limit the scale of exposure of the employees, to guarantee that the radiation exposure stills as reduced as is acceptably obtained, to assure that the limit values of radiation exposure are not overtaken, and to perceive not foreseen changes in the variables that affect employees’ radiation exposure. According to UNSCEAR, the total dose that a person is subjected to from Radon inhalation can vary between 0.2 and 10 mSv per year. 

### 3.2. Knowledge Assessment Survey on the Topic and on the Perception of the Risk Level 

The main difficulty in communicating the risk of exposure level to polluting agents or hazardous situations is the perception that those exposed have about the risk [92]. Often, the very notion of the risk of exposure and the danger of the situation varies depending on, for example, the level of education, and the information available, but mainly how this communication is made [93]. The assessment of this perception by those exposed, or potentially exposed to the risk, was determined by conducting a survey, made available through social networks such as FACEBOOK and LINKEDIN, which was made available for three months, from January 2021 to the end of March 2021. Thus, the target population covered by the two social networks, estimated for Portugal, is around 6 million users. In addition to the invitations to participate sent by the aforementioned social networks, with the request to share the link, to enhance its dispersion among the largest possible number of users. Requests were also sent for the dissemination of the questionnaire link to all national public and private higher education entities. Thus, it was intended to obtain a characterization of the knowledge on the topic, as well as to assess the degree of perception of the risk of exposure. The survey, presented in Table 1, is divided into three stages, which allow, firstly, the measurement of the knowledge on the theme, using an eliminatory stage, in response to the question “Have you heard about Radon?”, which just allows the progression to the next stage for respondents who answered affirmatively. For respondents who answer negatively, the survey moves on to the last questions, regarding the sociological characterization of the sample. In a second stage, a set of multiple-choice questions assess the degree of knowledge on the topic, as well as an existing understanding of the risk and danger of exposure to Radon gas. In the third stage, the understanding of how different levels of risk are communicated and, mainly, about the correlation between risk and exposure levels. This correlation is fundamental for the understanding of the dosages to which anyone can be exposed. Finally, the survey presents a set of questions of a personal, non-identifying nature, such as age, gender, educational qualifications, location of residence, and work, to be able to verify the levels of understanding of the subjects at a national level, and other aspects of sociological characterization. The conduction of this survey complied with the legal requirements provided for in Law no. 58/2019, of 8 August, which ensures the implementation, in the national legal order, of Regulation (EU) 2016/679 of the Parliament and of the European Council, of 27 April 2016, on the protection of individuals regarding the processing of personal data and on the free movement of such data. 

### 3.3. Radon Risk Management Framework Definition

The use of surveys to validate and justify a given tool is a methodology widely disseminated in several areas of knowledge, as demonstrated by the various works available [94]. In the specific case of Radon concentration and the perception that populations have of the risk of exposure, there are already some referenced works. For example, Pantelic et al. (2019) carried out a qualitative analysis of indoor air in Europe based on surveys to perceive opinions about the importance of the theme in European Union countries [95]. On the other hand, Vukotic et al. (2019) surveyed householders in Montenegro when placing passive detectors to assess the perception of populations on the subject of Radon exposure, and thus could serve as a basis for the national Radon action plan [96]. Dowdall et al. (2017) applied a survey to gather information about the construction of buildings and the perception that their householders have about the associated exposure risk, intending to develop a new survey protocol in Ireland [97]. Yarmoshenko et al. (2020) assessed Radon exposure by householders of new energy-efficient multi-story apartment houses in four Russian cities, namely, Ekaterinburg, Chelyabinsk, Saint-Petersburg, and Krasnodar [98]. As can be seen, surveys to assess the perception of exposure risk and to acquire data are widely used. In the case presented here, the survey is used to assess the degree of knowledge about the problem raised by Radon exposure and to assess the degree of perception of the risks associated with this exposure. It is an objective as well to evaluate how this exposure should be communicated. This communication of the risk of exposure to Radon to populations has been the object of analysis for many years, as shown by the works of Golding et al. (1992), Bostrom et al. (1992), and Fisher and Johnson (1990), reporting the difficulty in communicating a topic on which populations, in general, do not have significant literacy [99,100,101]. In fact, in line with other works on risk communication of other natures, where the perception of risk was higher, the greater the literacy on the subject [93,102,103,104,105,106]. Thus, the assessment of knowledge of populations using a survey is presented as necessary when one intends to develop a tool to quantify the risk and present it in a way that is understandable to the exposed population.

## 4. Results and Discussion

### 4.1. IRREI—Indoor Radon Risk Exposure Indicator

#### 4.1.1. Definitions and Concepts

Radon Exposure can be defined as the amount of time a person spends for any given Radon concentration [107] and estimated based on the knowledge of building occupancy, which specifies where and how individuals spend their time, along with knowledge of Radon activity concentrations in each indoor space [108]. Statistically, the Radon concentration data follow a log-normal distribution being its average concentration typically computed using the Geometric Mean (GM) and the Geometric Standard Deviation (GSD). It is important to note that a considerable number of studies and countries do not use GM and GSD and, therefore, to maintain comparability, it is recommended to present data using both the GM and Arithmetic Mean (AM) and their respective measures of Standard Deviation (i.e., GSD, SD) [10]. However, in this work, it was decided to use the AM and SD, since we will use dose conversion factors for Radon—that have been derived from epidemiological studies [109]—and are recommended by the United Nations Scientific Committee on the Effects of Atomic Radiation (UNSCEAR) [110,111].

Several Radon risk models in the literature share a common principle, i.e., the lifetime risk of Radon-induced lung cancer increases with increased exposure to Radon progeny [112]. Two of the most important ways to quantify the risk associated with Radon Exposure are:

- Excess Relative Risk (ERR): an epidemiological risk measure that quantifies how much the level of risk among persons with a given level of exposure exceeds the risk of non-exposed persons;
- Relative Risk (RR): represents the ratio of the probability of a disease occurring in the exposed group versus a non-exposed group [112]. ERR and RR are related through the equation:

ERR = RR − 1(1)

However, these models have been designed for the study of natural radiation epidemiological risk exposure, and their use needs background on epidemiological risk analysis for better understanding. Thus, in this section, the IRREI—Indoor Radon Risk Exposure Indicator, a simple and effective indicator for effective indoor Radon risk exposure communication will be put forward. The IRREI is based on a four-level scale computed upon prior knowledge of building occupancy along with knowledge of Radon activity during specific measurement intervals and mapped to an annual reference, i.e., the Indoor Annual Effective Dose. 

#### 4.1.2. Definition of Measurement Intervals 

Data collected with a continuous Radon detector can then be aggregated, based on conditional timestamped occupation, i.e., where only effective occupancy is considered using distinct periods. The time periods considered have been defined taking into account the approach presented by Lopes et al., where [46]:

- Real-Time (RT): represents the period that comprises the last hour. This metric is of great importance for Radon Risk Management since it can be used for making decisions that may include specific mitigation actions, such as triggering manual or mechanical ventilation mechanisms.
- Very Short-Term (VST): represents the occupied period that comprises the last 24-h;
- Short-Term (LT): represents the occupied period that comprises the last week;
- Long-Term (LT): represents the occupied period that comprises, at least the last 3 months;

The periods previously introduced will be the basis for the computation of several metrics regarding indoor effective exposure, and average Radon concentrations. In the next subsection, a reference metric called Indoor Annual Effective Dose (IAED) will be used to quantify the effective radiation dose in one year for a given occupancy profile.

#### 4.1.3. Indoor Annual Effective Dose (IAED)

To quantify the Indoor Annual Effective Dose (IAED) from Radon exposure, we followed the same approach presented in Chen & Moyer (2010) [108] by using the formula recommended by the United Nations Scientific Committee on the Effects of Atomic Radiation (UNSCEAR) in [110]:

IAED = 40 Bq·m^−3^ × 0.4 × 7000 h × 9 nSv·(Bq·m^−3^·h)^−1^ = 1.0 mSv (2)

In Equation (2), the value 40 represents the Annual Arithmetic Mean Radon Concentration (AAMRC) in the units of Bq·m^−3^, the value of 0.4 was used as the equilibrium factor (F-factor) for Radon indoors, a recommended value of 9 nSv (Bq·m^−3^·h)^−1^ was used to convert Radon equilibrium-equivalent concentration to population effective dose, and 7000 h represents the Annual Average Occupancy (AAO), i.e., 80% of occupancy. Note that the dose conversion factor 9 nSv (Bq·m^−3^·h)^−1^, as presented in [110], is still considered appropriate for average effective dose calculations. The F-factor is used to describe the ratio between Radon and its progeny. An F-factor of one means equal amounts of Radon and its progeny. An F-factor of 0.4 is taken as representative for the indoor environment and 0.6 is used for outdoors [110]. 

For a proper investigation of the occupancy impact in the IAED, we adapted Equation (1) to reflect both the AAMRC and the Daily Average Occupation (DAO) variations. The DAO is represented in hours and defined as AAO = 365 × DAO. Given this, Equation (3) can be rewritten as:

IAED = AAMRC (Bq·m^−3^) × 0.4 × 365 × DAO (h) × 9 nSv·(Bq·m^−3^·h)^−1^ (3)

#### 4.1.4. Definition of IRREI

To graphically represent the information, we opted to define an Indoor Radon Risk Exposure Indicator (IRREI) that will be represented using an integer scale with values between one and four. This scale is then referenced to an IAED of 1 mSv. The IRREI is an adimensional integer represented in the interval between one and four. The IRREI was designed to reflect a specific color code for Graphical User Interfaces (GUI) purposes. IRREI is represented as an integer value where one refers to an IAED below 1 mSv. The intervals and colors were defined based on the dose limits defined in [69] by the International Commission on Radiological Protection (ICRP):

- Green (IRREI = 1): represents an IAED less than or equal to 1 mSv/year, i.e., the dose limit defined in [69] for Public Exposure;
- Yellow (IRREI = 2): represents an IAED higher than 1 mSv/year and lower than 5 mSv/year, and reflects the dose limit recommended for Public Exposure in special situations, i.e., a higher value can be allowed in a single year if the average over 5 years does not exceed 1 mSv per year;
- Orange (IRREI = 3): represents an IAED between 5 mSv/year and 20 mSv/year, the limit imposed for Occupational Exposure;
- Red (IRREI = 4): represents an IAED above 20 mSv/year, i.e., above both Public and Occupational Exposure Limits.

These intervals and their correspondent colors will be the base of the visual data analytics approach to dynamically assess Radon Risk in a specific home or compartment by computing the Indoor Annual Effective Dose (IAED) metric based on two criteria that are dynamically changing over time, i.e., the Daily Average Occupation (DAO) profile and the Arithmetic Mean Radon Concentration (AAMRC).

**Figure 4:**
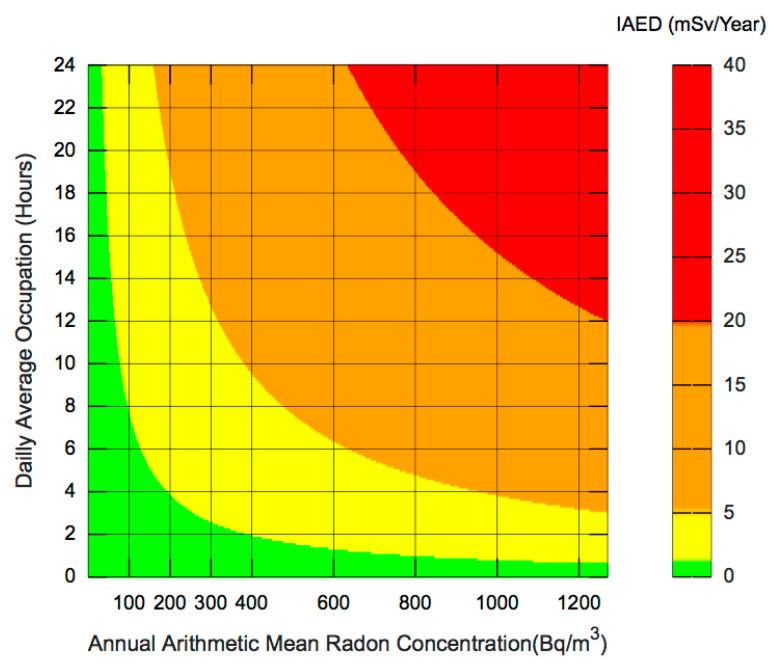
The IRREI abacus obtained using the Indoor Annual Effective Dose (IAED) as a function of the Annual Arithmetic Mean Radon Concentration (AAMRC) and the Daily Average Occupation (DAO).

Figure 4 depicts the IAED as a function of the AAMRC and the Daily Average Occupation (DAO), cf. Equation (2), in the interval between 0 and 1270 Bq·m^−3^, and 0 to 24 h, respectively.

Figure 4 can be used as a reference abacus, i.e., a graphical representation that allows a simpler risk assessment based on a priori occupancy schedules, i.e., Daily Average Occupation (DAO), and the continuously measured data obtained by IoT-based Radon detectors, that is then used to compute the Arithmetic Mean Radon Concentration (AMRC). 

For example, if we consider a building compartment with a DAO of 8 h, and an AMRC of 300 Bq·m^−3^ for a specific time, as defined in the previous section, we can calculate the IED and the AMRC for that specific period, and if both conditions are maintained during one year, we can extrapolate for one year and compute the IRREI, through the simple overlay of DAO and AMRC data from a specific time window (RT, VST, ST or LT) directly on the abacus. In this example, if we consider a Short-Term time, we can compute the ST-IED, which represents, by juxtaposition with the abacus, an ST-IRREI level equal to two, i.e., yellow.

#### 4.1.5. IoT-Oriented Methodology for Radon Risk Management

**Figure 5:**
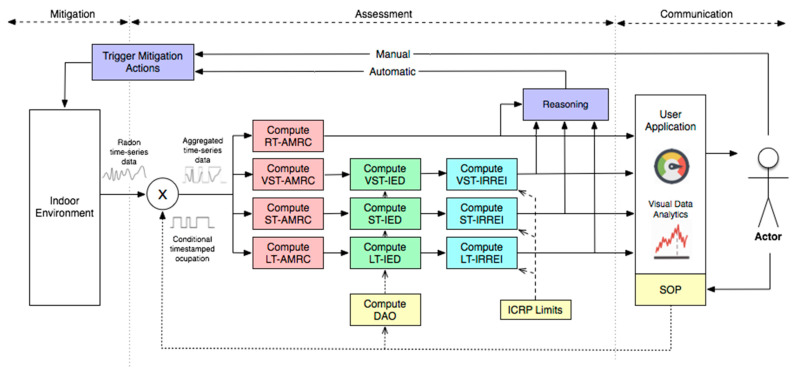
The IoT-oriented methodology for Radon risk management based on IRREI, where the abbreviations represent: RT-AMRC, Real-Time Average Mean Radon Concentration; VST-AMRC, Very-Short-Term Average Mean Radon Concentration; ST-AMRC, Short-Term Average Mean Radon Concentration; LT-AMRC, Long-Term Average Mean Radon Concentration; VST-IED, Very-Short-Term Indoor Effective Dose; ST-IED, Short-Term Indoor Effective Dose; LT-IED, Long-Term Indoor Effective Dose; VST-IRREI, Very-Short-Term Indoor Radon Risk Exposure Indicator; ST-IRREI, Short-Term Indoor Radon Risk Exposure Indicator; LT-IRREI, Long-Term Indoor Radon Risk Exposure Indicator; DAO, Daily Average Occupation; and SOP, Specific Occupation Period.

Figure 5 presents an analytics methodology for continuous Radon risk management that is divided into three main domains: (1) risk mitigation; (2) risk assessment, and (3) risk communication. The proposed methodology was designed for the IoT age, where IoT devices continuously collect real-time Radon time-series data that are then aggregated based on the timestamped Specific Occupation Period (SOP), defined by the building manager, for a specific compartment. Radon time-series data are then aggregated to compute the short-term (ST) and long-term (LT) Average Mean Radon Concentration (AMRC). Then, based on the calculus of the Daily Average Occupation (DAO), the Indoor Effective Dose (IED) is obtained for the following three-time windows VST, ST, and LT. Finally, and based on the abacus intro introduced in the previous section, the IRREI is computed also for VST, ST, and LT. These metrics, VST-IRREI, ST-IRREI, and LT-IRREI are then reasoned in conjunction with the continuous RT-AMRC metric, and, as a result, specific mitigation actions may be triggered manually or automatically. 

### 4.2. Survey Results 

#### 4.2.1. Characterization of the Population and the Sample 

**Figure 6:**
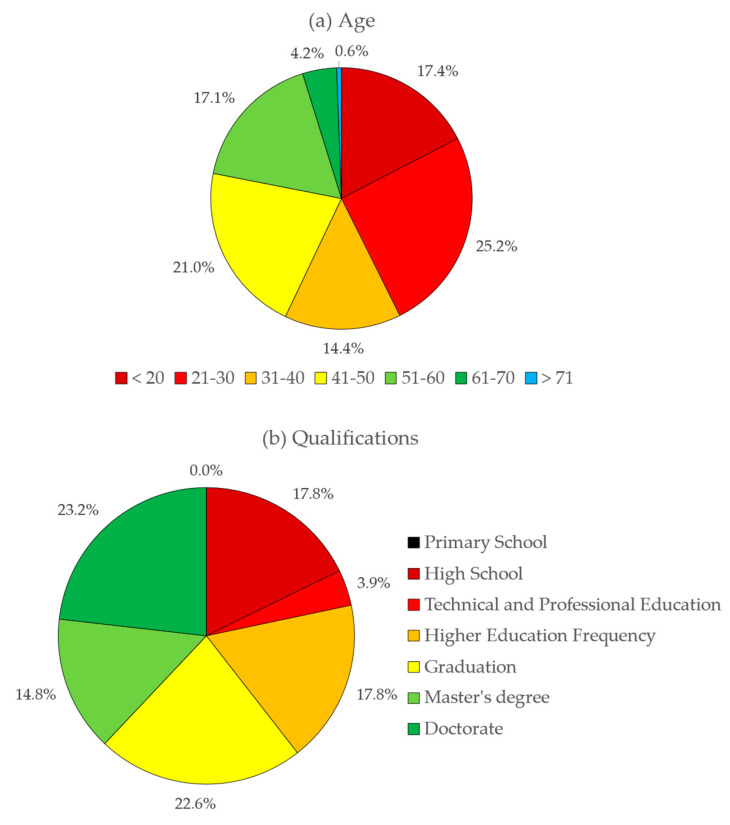

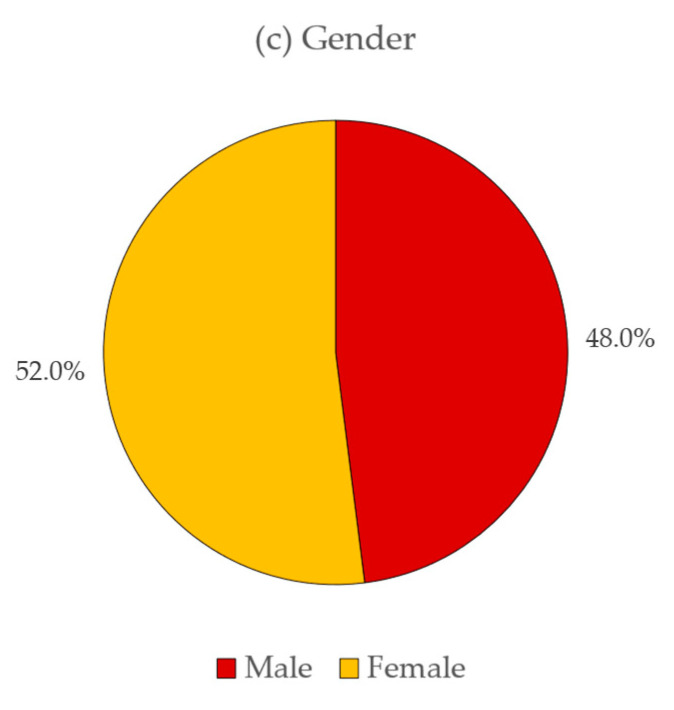
Characterization of the sample: (**a**) age; (**b**) qualifications; and (**c**) gender.

The survey was active during the period from 5 January to 16 March 2021, with a total of 873 valid responses. This set, considering a population of 6 million individuals, corresponding to the Portuguese users of the social networks FACEBOOK and LINKEDIN, for a degree of confidence of 95%, allows calculation of a margin of error for the survey carried out, as being 3%. In other words, this margin of error, or confidence interval, allows the affirmation that the results of this survey represent the real opinions of the population since the error is only 3%. Of the results obtained, 17.4% correspond to the age range equal to or below 20 years old, 25.2% correspond to the age range between 21 and 30 years old, 14.4% correspond to the age range between 41 and 50 years old, 17.1% correspond to the age range between 51 and 60 years old, 4.2% correspond to the 61 and 70 years old age range, and 0.6% correspond to the equal or older than 71 years old age range. As can be seen from the results obtained, the levels corresponding to young adults and adults present a higher participation rate, totalizing 78% of the total of respondents, which is in line with the results presented for studies carried out with similar tools, as described in the studies conducted by Lange et al. (2017) and Moretti et al. (2020) [112,113]. Concerning the gender of the respondents, the distribution shows a tendency towards an equal division among the respondents, with the male gender presenting a slight increase, with 52%, while the female gender represents 48% of the total responses. This result is also in line with previous studies, where male participation presents slightly higher values, although there is currently a trend towards the approximation between genders. Regarding the educational qualifications of the sample, there is a distribution of 17.7% for those who attend or hold complete secondary education, 3.9% for those who attend or hold technical–vocational courses, 17.7% for those who attend higher education, 22.5% for graduates, 14.7% for holders of a master’s degree and 23.1% for those with a doctorate. As can be seen, the level of academic qualifications presented by the sample, showing 78.5% of respondents with university qualifications, including those with a doctorate, the highest, does not represent the level of qualifications of the Portuguese population. In fact, according to the indicators presented on the PORDATA platform, which provides statistical data on Portugal, the percentage of individuals of both genders, aged between 18 and 64 years old is 25.4%. However, the fact that Portuguese public and private higher education entities were asked to distribute the survey by their email lists justifies this higher level of academic qualifications presented in the study. Based on the results obtained, and despite the high level of academic qualifications presented, there is a high degree of ignorance on the topic, as will be seen in the results obtained in the survey responses (Figure 6). Concerning the geographical distribution of the origin of the answers, those originated from all districts of mainland Portugal, but also in the autonomous regions of the Azores and Madeira. It was also found that the districts that provided the largest number of responses were those that, in some way, are related to the occurrences of Radon, more specifically, the districts of Viana do Castelo, Braga, Porto, Vila Real, Viseu, and Guarda, on the mainland, and the district of Angra do Heroísmo, in the autonomous region of the Azores. This participation from these districts may be related to the fact that the theme is more widespread, as these are regions presented in the Radon Map, carried out by ITN—Instituto Tecnológico e Nuclear, integrated into IST—Instituto Superior Técnico, corresponding to the areas of greatest incidence. 

#### 4.2.2. Results Obtained in Step 1 of the Survey

**Figure 7:**
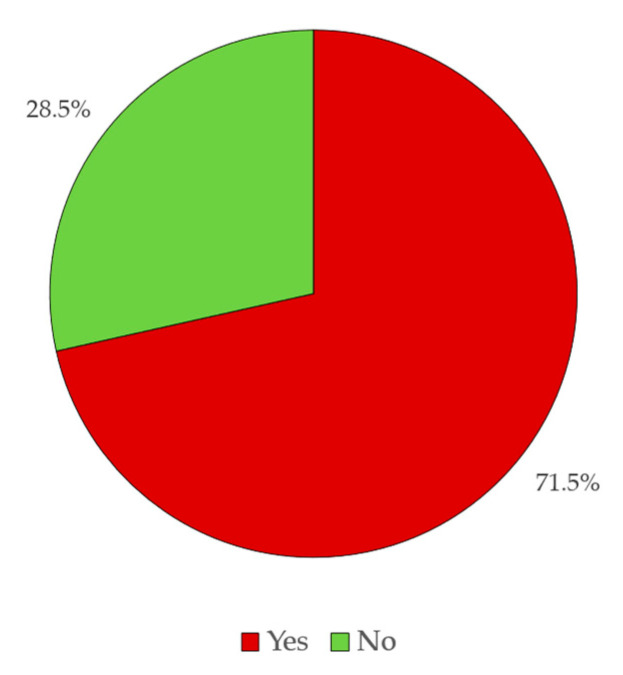
Results obtained in Step 1.

Of all the responses gathered, it was found that 71.5% of the respondents answered negatively to the question “Have you heard of Radon?”, while only 28.5% answered it affirmatively. Although, as was verified in the previous section, the level of academic qualifications is high, there is a high level of ignorance regarding the concepts and definitions on the theme that, as will be shown below, hinder the perception of the level of knowledge about risk and danger of exposure to Radon. In fact, a total of 71.5% of the respondents revealed some sort of ignorance on the topic, making evident that the communication of risks and dangers of exposure to Radon has to be carried out in a very pedagogical way, and cannot be limited to the simple presentation of the level of risk or potential for danger, since the receptors are not prepared to understand the information (Figure 7).

#### 4.2.3. Results Obtained in Step 2 of the Survey 

**Figure 8:**
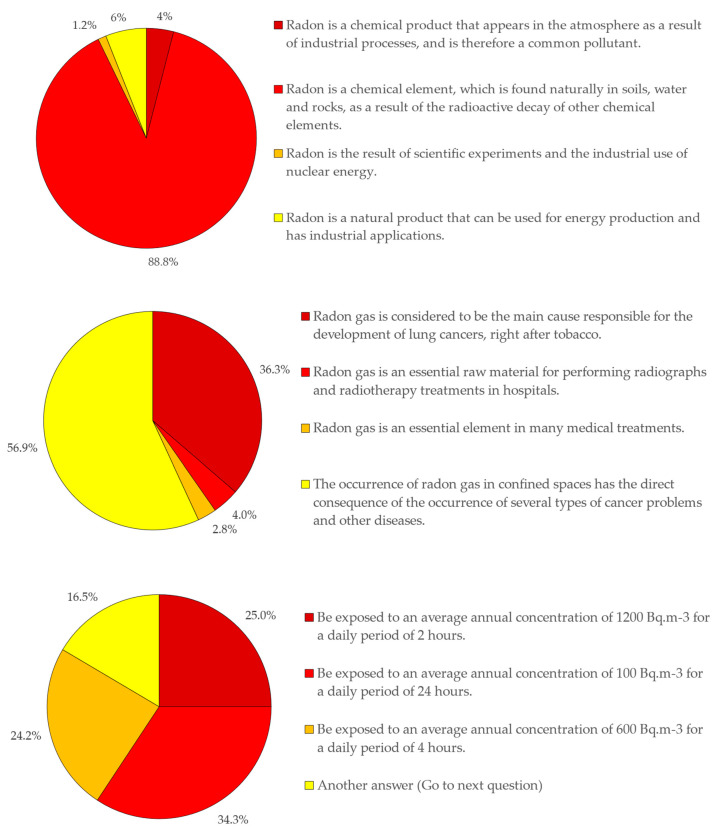
Results extracted from Step 2.

The results obtained for the questions in Step 2 of the survey, which aim to assess the levels of knowledge for respondents who answered affirmatively to the question presented in Step 1, demonstrated that respondents who know the topic have a concrete notion regarding the origin of Radon gas, with 88% of respondents confirming that “Radon is a chemical element that is found naturally in soils, water, and rocks, as a result of the radioactive decay of other chemical elements”. Regarding the relationship of Radon with diseases, the majority of respondents, 56.9%, related exposure to Radon with the occurrence of various diseases, whether of an oncological nature or not, while 36.3% directly relate the exposure to Radon gas with lung cancer. Despite some flaws in the accuracy of the correlation of exposure to Radon gas with the type of associated diseases, it appears that respondents, mostly, relate exposure to Radon gas with a set of diseases, so it is common sense the cause–effect correlation between exposure and disease. The greatest difficulty arises precisely in quantifying the dose. That is the correlation between the amount of gas to which one is exposed and the duration of that exposure. This difficulty is found in the distribution of the answers obtained for Question 4, where similar levels of exposure are presented, but which led to different analyses by the respondents, with some attributing greater importance to the concentration of the gas, 25%, while others at different exposure times, with 34.3% and 24.2%, respectively. Only 25% of respondents answered that it could be a similar level of exposure, thus demonstrating an ability to correctly read the information presented (Figure 8). 

#### 4.2.4. Results Obtained in Step 3 of the Survey 

**Figure 9:**
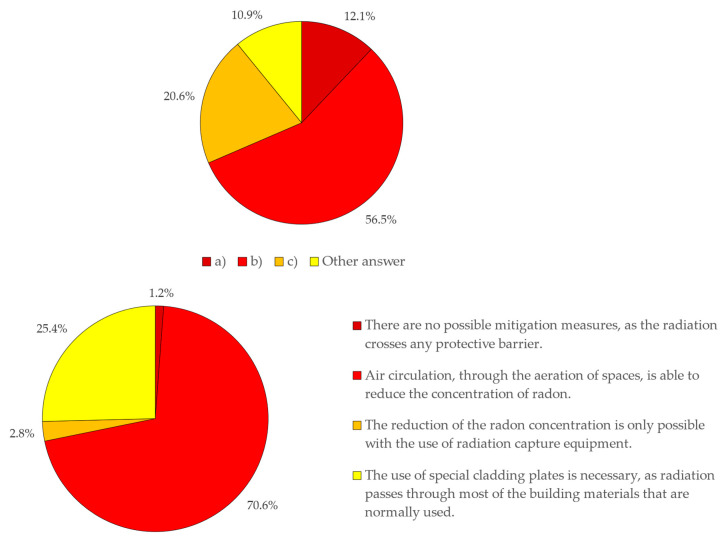
Results extracted from Step 3.

If, on the one hand, knowledge about Radon gas and associated risks exists, concerning the risk exposure, this perception is conditioned on how risk communication is made, but mainly due to established stereotypes. Graphic communication systems, using color patterns, such as blue–red colors, symbolizing hot and cold, or green–red colors, symbolizing low risk or high risk, have long been established and are part of knowledge and common sense. The results obtained in the analysis of Question 5 show that, although the figures presented the same level of risk, 56.5% of the respondents opted for option (b), which presents the point corresponding to the level of risk in the red zone, followed by answer (c), with 20.6%, which presents the point corresponding to the level of risk in the yellow zone. Answer (a), which corresponds to the level of risk coinciding with the green zone, with 12.1%, was the third most chosen answer, most likely since respondents “suspected of” the existence of a trap and chose the opposite sense to what would be expected. Finally, option (d), “Other responses”, follows, with 10.9%, and, of these respondents, a majority argued that the levels of risk presented are identical, demonstrating that they understood the concept of dose and capacity to understand the correlation between exposure time and gas concentration. Regarding the perception of the mitigation measures and the forms that can be used, it appears that the respondents answered mostly, with 70.6%, in a correct way, identifying that the air circulation, through aeration of the spaces, as being the most effective measure to reduce the concentration of Radon in confined spaces (Figure 9). 

#### 4.2.5. Communication of Risk Levels 

From the results obtained, it can be inferred that a tool for communicating the risk of exposure to Radon, which includes the fundamental factors that lead to the determination of the dosage, is a determining aspect for the understanding by the public in general, of the results of the performed diagnoses. This communication is more important if it has a pedagogical character, allowing the receptors of the information, by itself, to analyze and compare different results, to verify how they can be affected by more or less favorable situations, and changing the factor which they can have direct control, such as the time of exposure, but also, as was demonstrated by the responses obtained on the perception of mitigation measures, by the adoption of procedures that allow the reduction of the concentration of Radon gas in the permanence space. The adoption of an intuitive use tool, developed according to the green–yellow–orange–red color code, indicating levels of, respectively, very low risk, low risk, moderate risk, and high risk, is a disruptive milestone regarding the transmission of information in a clear, understandable way and, mainly, minimizing the alarmism caused by an unknown situation. The annual indoor effective dose (IAED), expressed in mSva·year^−1^, can be presented graphically as a function resulting from the relationship between the annual arithmetic mean Radon concentration (AAMRC), expressed in hours, and the daily average occupation (DAO), expressed in Bq·m^−3^. This relationship, which is shown in Figure 4, allows the presentation of the results obtained in the diagnostic evaluations, indicating expeditiously, both to the users of the space in question, but also to the managers of that same space, to take all the necessary decisions so that the risk levels of exposure to Radon remain at the recommended values. This analysis can, for example, starting from an initial evaluation, and by changing the parameters, such as AAMRC and DAO, to verify the evolution of the IAED, justifying the adoption of certain measures to the detriment of others, or even justifying the realization of investments, for example, for the installation of continuous monitoring equipment, or means of active and passive protection in buildings.

## 5. Conclusions

The implemented research has developed an IoT-oriented evaluation tool for Radon Risk Management (RRM), based on the design of a simple and intuitive Indoor Radon Risk Exposure Indicator (IRREI), purpose-built to be employed as an aiding decision-making tool for building owners, building designers, and buildings managers, or simply as an alert flag for the ordinary citizen problem awareness. 

The proposed methodology was designed for graphic representation aligned with the requirements of the current IoT age, i.e., the methodology is robust enough for continuous data collection with specific spatial-temporal attributes and, therefore, a set of adequate Radon risk-related metrics can be extracted and proposed. 

Metrics are summarized taking into account the application case, taken as a case study for data validation, by including relevant variables to frame the study, such as the regulatory ICRP dosimetric limits, building occupancy (spatial dimension), and occupant exposure periods (temporal dimension). 

The main conclusions of this investigation are the following;

(1) To quantify the risk of exposure to a certain hazard and to straightforwardly explain that it is mandatory, in a preliminary stage, to assess people’s awareness of the problem by implementing a specific survey.
(2) The implemented survey was designed to assess awareness towards Radon risk exposure, and it was divided into three complementary stages: firstly, it evaluated the common knowledge about Radon risk exposure, secondly, it assessed the depth of knowledge on the topic and its consequences concerning human health, and thirdly it evaluated the understanding on how different risk level can be communicated and, mainly, on the correlation between Radon risk and growing exposure levels.
(3) The IRREI—Indoor Radon Risk Exposure Indicator is a simple and effective indicator for effective indoor Radon risk exposure communication. The IRREI is based on a 4-level scale—green (IRREI = 1), yellow (IRREI = 2), orange (IRREI = 3), and red (IRREI = 4)—computed upon prior knowledge of building occupancy along with knowledge of Radon activity during specific measurement intervals and mapped to an annual reference, i.e., the Indoor Annual Effective Dose.
(4) Based on IRREI calculation, an analytical methodology for continuous Radon risk management divided into three main domains: (1) risk mitigation; (2) risk assessment, and (3) risk communication was conceived. The proposed methodology was designed for the IoT age, where IoT devices continuously collect real-time Radon time-series data that are then aggregated based on the timestamped Specific Occupation Period (SOP), defined by the building manager, for a specific room or compartment.
(5) Based on survey results, it can be concluded that a tool for communicating the Radon exposure risk is more effective when it is implemented in an intuitive use tool, i.e., developed according to the green-yellow–orange-red color code, indicating levels of, respectively, very low risk, low risk, moderate risk, and high risk.
(6) The graphical representation of IRREI is a disruptive milestone regarding the transmission of Radon risk information in a clear, understandable way and, mainly, minimizing the alarmism caused by an unknown situation.

## Figures and Tables

**Table 1:** Survey presented through GOOGLE FORMS to assess the perception of risk associated with exposure to Radon.

Eliminatory Question			
Have you heard of Radon?		Yes	No
		*Go to the next section*	*Survey ends*
**Introduction**			
The present study aims to analyze the knowledge about the existence and effects of Radon, mainly in closed spaces, through the evaluation of the concepts disseminated and transmitted essentially by the media, and the current state of perception of them by the potentially exposed populations.			
**I. Assessment of knowledge on the topic**			
Of the following statements, please indicate which one is true:			
Radon is a chemical product that appears in the atmosphere as a result of industrial processes and is, therefore, a common pollutant. Radon is a chemical element, which is found naturally in soils, water, and rocks, as a result of the radioactive decay of other chemical elements. Radon is the result of scientific experiments and the industrial use of nuclear energy. Radon is a natural product that can be used for energy production and has industrial applications.			
*Go to the next section*			
**II. Evaluation of knowledge about the health effects of Radon gas**			
Of the following statements, please indicate which one is true:			
Radon gas is considered to be the main cause responsible for the development of lung cancers, right after tobacco. Radon gas is an essential raw material for performing radiographs and radiotherapy treatments in hospitals. Radon gas is an essential element in many medical treatments. The occurrence of Radon gas in confined spaces has the direct consequence of the occurrence of several types of cancer problems and other diseases.			
*Go to the next section*			
**III. Assessment of the perception of the degree of exposure**			
From the following statements, please indicate which one represents the highest degree of exposure to Radon gas:			
Be exposed to an average annual concentration of 1200 Bq·m^−3^ for a daily period of 2 h. Be exposed to an average annual concentration of 100 Bq·m^−3^ for a daily period of 24 h. Be exposed to an average annual concentration of 600 Bq·m^−3^ for a daily period of 4 h. Another answer (Go to next question)			
*Go to the next section*			
If you answered “Another answer”, what would it be?			
*Go to the next section*			
**IV. Assessment of the perception of the degree of risk and the traditional conditions of communication**			
From the following images, indicate which one corresponds to the level of greatest risk for those who are exposed to the conditions indicated by the black dot:			
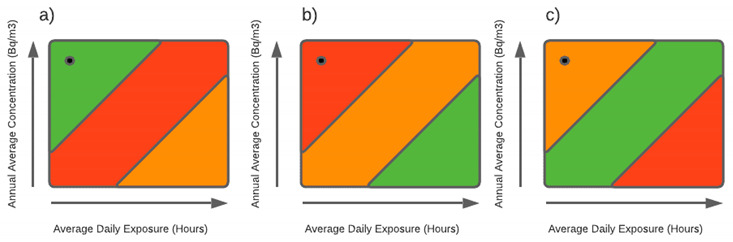			
Note: Red—High risk; Yellow—Medium risk; Green—Low risk			
(a)	(b)	(c)	Another answer (Go to next question)
If you answered “Another answer”, what would it be?			
*Go to the next section*			
**V. Assessment of the perception of the existence of mitigation measures**			
From the following images, indicate which mitigation measure is capable of reducing the concentration of Radon inside a home or workplace:			
There are no possible mitigation measures, as the radiation crosses any protective barrier. Air circulation, through the aeration of spaces, can reduce the concentration of Radon. The reduction of the Radon concentration is only possible with the use of radiation capture equipment. The use of special cladding plates is necessary, as the radiation passes through most of the building materials that are normally used.			
*Go to the next section*			
**Analysis of the population participating in the survey:**			
Ages	<20; 21–30; 31–40; 41–50; 51–60; 61–70; >71		
Qualifications	Basic education; 12th year; higher education attendance; graduation; master’s degree; doctorate		
Place of residence			
Gender	Male; Female		
*Finish the survey and submit*			

## Data Availability

The data presented in this study are available on per request to the corresponding author.
